# Comparative Efficacy of Reused Medium Cut-Off Dialyzers on Uremic Toxin and Cytokine Clearance: A Randomized Controlled Trial

**DOI:** 10.3390/life15091468

**Published:** 2025-09-18

**Authors:** Eakalak Lukkanalikitkul, Nichnan Jirayuphat, Sirirat Anutrakulchai

**Affiliations:** 1 Center of Excellence in Kidney Diseases, Faculty of Medicine, Khon Kaen University, Khon Kaen 40002, Thailand; 2 Division of Nephrology, Department of Internal Medicine, Faculty of Medicine, Khon Kaen University, Khon Kaen 40002, Thailand; nun49er69@hotmail.com (N.J.); sirirt_a@kku.ac.th (S.A.)

**Keywords:** reused dialyzer, medium cut-off dialyzer, middle molecule uremic toxin

## Abstract

Introduction: Expanded hemodialysis using medium cut-off (MCO) dialyzers effectively removes middle-molecule uremic toxins, comparable to hemodiafiltration, but their single-use designation increases the dialysis costs. This study evaluated the efficacy and safety of reusing two MCO dialyzers available in Thailand. Methods: In this randomized controlled trial, hemodialysis patients were assigned to receive treatment with either Theranova^®^ 500 or Elisio^®^ 21HX dialyzers. Each dialyzer was reprocessed using peracetic acid and reused for up to 15 sessions. Dialyzer performance was assessed by the reduction ratios (RRs) of solutes, including β2-microglobulin (β2-MG), kappa and lambda free light chains (κ-FLC, λ-FLC), and interleukin-6 (IL-6), at baseline and the 2nd, 5th, 10th, and 15th sessions. Results: Forty-eight patients were enrolled (mean age 63.6 ± 13.7 years; 62.5% male) and randomized into 2 groups with comparable baseline characteristics. RRs for β2-MG, κ-FLC, and λ-FLC were similar between the groups and declined modestly over time after dialyzer reused (β2-MG: 78.2% to 72.5% vs. 77.2% to 74.5%, κ-FLC: 64.6% to 51.3% vs. 58.9% to 49.5%, and λ-FLC: 51.2% to 46.4% vs. 49.4% to 39.2% in the Theranova^®^ 500 and Elisio^®^ 21HX groups, respectively). Theranova^®^ 500 demonstrated significantly higher IL-6 clearance in the 2nd (29.9% vs. 16.0%; *p* = 0.018) and 5th (23.8% vs. 7.7%, *p* = 0.031) sessions. It also showed a non-significant trend toward lower dialyzer survival (HR 3.98; *p* = 0.085) and higher, though clinically acceptable, albumin loss (mean difference 0.56 g/session; *p* < 0.001), which decreased with reuse. Conclusions: Both MCO dialyzers demonstrated comparable overall performance during reuse. Theranova^®^ 500 provided better IL-6 clearance with manageable albumin loss. Implementation of high-quality dialyzer reuse protocols may optimize clinical efficacy and patient outcomes while balancing cost, accessibility, and environmental considerations.

## 1. Introduction

Cardiovascular disease and infections remain the leading causes of mortality in patients with end-stage kidney disease (ESKD) receiving hemodialysis (HD) [1]. Middle-molecule uremic toxins and inflammatory cytokines, inadequately cleared by conventional HD, are important contributors to these outcomes [2]. Uremic toxins are classified by molecular weight into small molecules (<500 Da), middle molecules (500–60,000 Da), and protein-bound toxins (PBUTs). Middle molecules are further subcategorized into small (500–15,000 Da), medium (15,000–25,000 Da), and large (25,000–58,000 Da) types [3]. To improve clearance of larger uremic toxins, medium cut-off (MCO) dialyzers with larger pore sizes have been developed. Expanded hemodialysis (HDx), which utilizes MCO dialyzers, has demonstrated superior removal of middle molecules and inflammatory cytokines compared to conventional high-flux HD [4]. This enhanced clearance is associated with improved quality of life and fewer dialysis-related symptoms [5].

**Figure 1:**
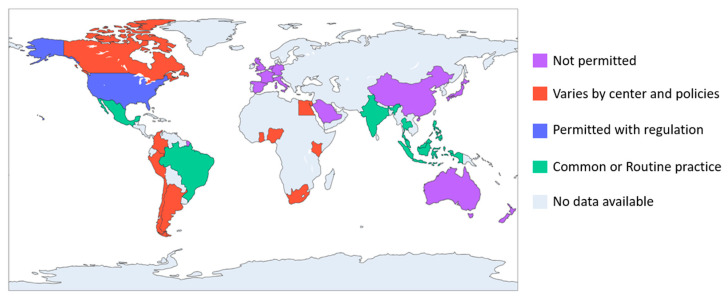
Global variation in clinical practices of dialyzer reuse in hemodialysis. This figure was generated by the authors based on publicly available data and guidelines [7,8,9,10,11,12,13,14,15,16,17,18,19,20]; no copyrighted material was used.

Despite these advantages, MCO dialyzers are typically designated for single use due to limited data on reuse safety and efficacy. However, dialyzer reuse can reduce costs and support environmental sustainability. Hemodialysis has a substantial carbon footprint, with 37% of emissions linked to equipment production and disposal [6]. In some settings, such as the United States, dialyzer reuse is common, driven by both economic and environmental concerns. Reuse beyond 10 sessions can significantly lower costs and waste [6]. The current practice of dialyzer reuse varies considerably across regions and is largely influenced by healthcare policies. Although the prevalence of dialyzer reuse has declined over time due to concerns regarding adverse outcomes, particularly bacterial bloodstream infections, it remains a common practice in many low- to middle-income countries, where more than 90% of patients undergo dialyzer reuse as a cost-saving measure with acceptable infection (Figure 1) [7,8,9,10,11,12,13,14,15,16,17,18,19,20].

Chemical disinfectants used in dialyzer reprocessing may alter membrane integrity and reduce fiber volume, potentially impairing clearance of uremic toxins and leading to albumin loss via the dialysate [11,21,22]. Safe reuse depends on standardized reprocessing protocols, including cleaning, rinsing, and testing total cell volume (TCV) [23,24]. A reduction in TCV > 20% from baseline is indicative of >10% reduction in small-molecule clearance efficiency and is generally considered unacceptable for continued reuse [25]. From a patient-care perspective, the evaluation of dialyzer performance is the responsibility of a multidisciplinary dialysis management team. Nephrologists and hemodialysis nurses play pivotal roles in the delivery of dialysis, the monitoring of reprocessed dialyzers, and the enforcement of safety protocols, thereby minimizing the risk of treatment inefficiencies or adverse outcomes associated with dialyzer reuse [26]. Therefore, if MCO dialyzers can be safely reused without performance loss, this could improve cost-effectiveness and access to high-quality dialysis.

## 2. Materials and Methods

### 2.1. Study Design and Population

This single-center, prospective, open-label randomized controlled trial was conducted at Srinagarind Hospital, Khon Kaen University, Thailand, between January and March 2024. Eligible participants were adults aged 18–80 years undergoing maintenance HD for >3 months with adequate dialysis (spKt/V > 1.2 for thrice-weekly, >1.8 for twice-weekly HD). All participants used either an arteriovenous fistula (AVF) or an arteriovenous graft (AVG) as their vascular access. Exclusion criteria included pregnancy, vascular access dysfunction, contraindications to dialyzer reuse (e.g., hepatitis B, HIV, multidrug-resistant organisms), advanced cancer or cardiovascular disease, life expectancy < 1 year, recent severe infections or hospitalization, immunosuppressive therapy, or history of severe dialyzer allergy. This study followed the CONSORT 2025 reporting guidelines (Supplementary Material, CONSORT checklist) [27].

### 2.2. Sample Size and Calculation

Sample size was based on repeated measures of reduction ratios (RRs) of middle-molecule toxins and cytokines over five timepoints (sessions 1st, 2nd, 5th, 10th, and 15th). Two groups (Theranova^®^ 500 vs. Elisio^®^ 21HX) were compared using one-way repeated measures ANOVA (G*Power v3.1.9.7), with partial eta-squared = 0.03, α = 0.05, power = 0.80, two groups, five repeated measurements, and a correlation = 0.7. To accommodate a 20% dropout, 24 participants per group (total *n* = 48) were enrolled.

### 2.3. Study Intervention

**Figure 2:**
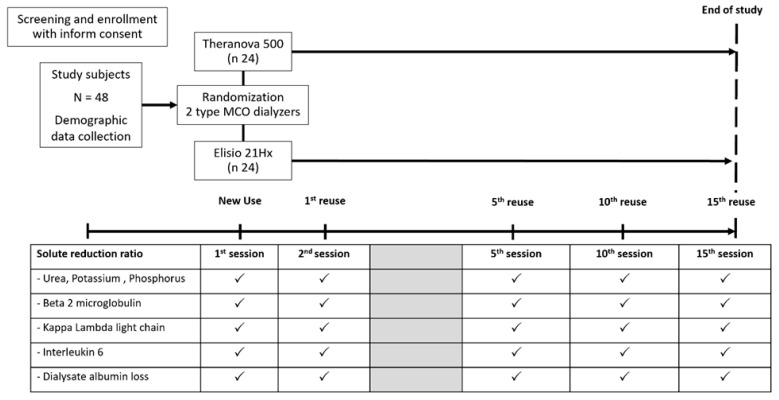
Demonstration of the study protocol.

Participants were randomized in a 1:1 ratio to receive HD using either Theranova^®^ 500 (Baxter International Inc., Deerfield, IL, USA) or Elisio^®^ 21HX dialyzers (Nipro Medical Co., Akita, Japan) (Figure 2 and Figure S1). Randomization was performed using a computer-generated stratified randomization sequence, stratified by age, sex, ESKD etiology, dialysis frequency, vintage, and vascular access. Allocation concealment was prepared by an independent statistician not involved in patient care. Characteristics of the study dialyzers are summarized in Supplementary Table S1.

Each participant underwent 4 h sessions, twice or thrice weekly, per their existing prescription, including individualized blood and dialysate flow rates, dialysate composition, intradialytic anticoagulation, and target dry weight. All dialysis was performed using Fresenius^®^ 4008H machines (Fresenius Medical Care, Bad Homburg vor der Höhe, Germany) with ultrapure water (microbial count < 0.1 CFU/mL; endotoxin < 0.03 EU/mL) [28]. Dialysis water and dialysate were monitored monthly during the study. The dialysis water and dialysate consistently met the quality standards for standard dialysis water and ultrapure dialysate, respectively (Supplementary Table S2). Intradialytic monitoring and management of complications were performed by trained HD nurses and nephrologists.

The intervention continued and repeated RRS of solutes were determined as shown in Figure 1 until either the 15th dialysis session was completed, or the assigned dialyzer was deemed unsuitable for reuse. Hemodialysis machines were disinfected with Citrosteril^®^ (Fresenius Medical Care, Bad Homburg vor der Höhe, Germany) in combination with a heat disinfection program (chemo-thermal disinfection) for each hemodialysis session. A 4% peracetic acid solution was used as the chemical disinfectant, with validated sterilization storage duration and thorough rinsing performed to ensure complete removal of residual disinfectant prior to patient use [21,22]. The standard dialyzer reprocessing protocol at Srinagarind Hospital adhered to the quality standards outlined in ANSI/AAMI RD47:2020 (detailed in Supplementary Materials) [24]. These guidelines encompass personnel qualifications and safety, equipment requirements, approved reprocessing materials, standardized procedures for reprocessing and storage, the disposition of rejected dialyzers, and preparation for subsequent use [24].

Both dialyzers were reprocessed using an automatic machine with 4% peracetic acid. If clots or abnormalities were detected, manual reassessment and rinsing with reverse osmosis water were performed. Dialyzers with >20% total cell volume (TCV) loss or unresolved clotting were considered non-reusable and discarded.

### 2.4. Outcomes

The primary objective was to compare the clearance efficiency of uremic toxins and inflammatory cytokines between Theranova^®^ 500 and Elisio^®^ HX21 dialyzers at initial use and at the 2nd, 5th, 10th, and 15th dialysis sessions. Efficacy was assessed by pre- and post-dialysis RRs of solutes spanning low to middle molecular weights: homocysteine (135 Da), phosphorus (30 Da), urea (60 Da), creatinine (113 Da), C-reactive protein (CRP, 115,000 Da), parathyroid hormone (PTH, 9400 Da), beta-2 microglobulin (β2-MG, 11,800 Da), kappa free light chain (κ-FLC, 22,500 Da), interleukin-6 (IL-6, 24,500 Da), and lambda free light chain (λ-FLC, 45,000 Da) while the secondary objectives were comparisons of solute clearance across repeated uses and between-group differences in dialysate albumin loss over time. Safety outcomes involved monitoring for dialyzer-related complications, including allergic reactions, membrane rupture, and intradialytic clotting.

### 2.5. Data Collection and Definition of Variables

Baseline demographic and clinical characteristics—including age, sex, weight, height, body mass index (BMI), comorbidities, etiology of ESKD, physical status, and socioeconomic factors—were obtained through patient interviews, physical examinations, and medical record reviews. Baseline laboratory measurements were collected two weeks prior to study initiation.

The RR was calculated by the following equations according to Bergström and Wehle [29].

RR (%) = [1 − (cCpost/Cpre)] × 100 Where cCpost = Cpost/[1 + (∆BW/0.2(BWpost))](1)

Cpre and Cpost represent the pre- and post-dialysis concentrations of the respective solutes. cCpost denotes the corrected Cpost adjusted for hemoconcentration. ΔBW refers to the reduction in body weight during the HD session, while BWpost indicates the body weight immediately after dialysis. The loss of albumin from dialysis was calculated by measuring the albumin quantity in dialysate samples collected at initial, 60 min, 120 min, and at the end of dialysis sessions by the following equation [30,31].

Kovr = M_D_/AUC Where M_D_ = C_DS_ × (Q_D_ × T_D_ = V_UF_)

Kovr is the overall clearance, M_D_ is the total mass removed, AUC is area under the dialysate albumin concentration–time curve, C_DS_ is the dialysate albumin concentration, Q_D_ is programmed dialysate flow rate, T_D_ is HD treatment duration and V_UF_ is actual ultrafiltration volume.

Serum concentrations of solutes were determined using standardized laboratory techniques: molecular absorption spectrometry was employed for urea and creatinine; immunoturbidimetric assay was used for β2-MG and CRP; electrochemiluminescence immunoassay was applied for IL-6; enzymatic assay was utilized for homocysteine; and the latex particle-enhanced immunoturbidimetric method was used to measure κ-FLC and λ-FLC.

### 2.6. Statistics

Continuous variables were expressed as mean ± standard deviation (SD) or median with interquartile range (IQR), and categorical variables as counts and percentages. Group comparisons used Student’s *t*-test or Mann–Whitney U test for continuous data, and Chi-square or Fisher’s exact test for categorical variables. Longitudinal outcomes were analyzed using repeated-measures ANOVA and the generalized estimating equation (GEE) model. Dialyzer survival was evaluated using Kaplan–Meier analysis and Cox proportional hazards models. Analyses were performed in STATA 17.0 (Stata Corp, College Station, TX, USA), with a two-tailed *p*-value of <0.05 considered statistically significant.

## 3. Results

Forty-eight patients were enrolled (mean age 63.6 ± 13.7 years; 62.5% male), with diabetic nephropathy as the leading ESKD cause (54.2%). Most (85.4%) used AVF access with mean dialysis vintage was 52.6 ± 32.8 months and mean baseline spKt/V per session was 1.9 ± 0.3. Baseline characteristics and prescriptions were comparable between groups (Table 1).

**Figure 3:**
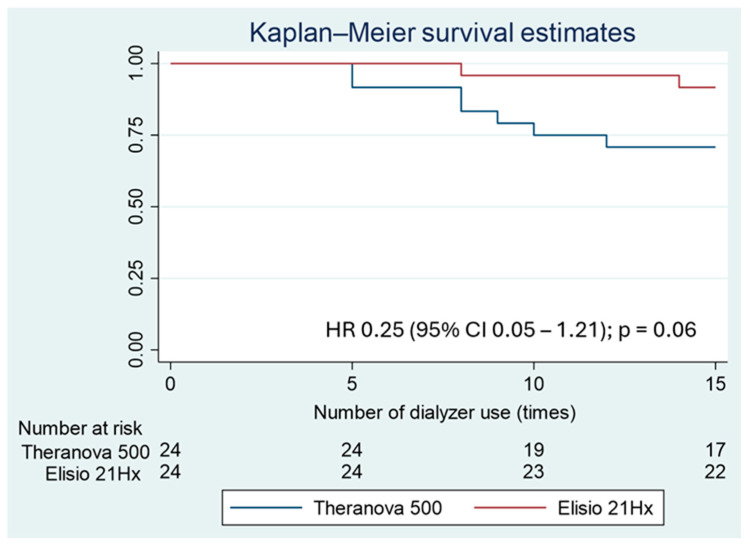
Survival of reprocessing medium-cutoff dialyzers compared between the Theranova^®^ 500 and Elisio^®^ 21HX groups.

Both groups received comparable dialysis prescriptions, including treatment duration, blood flow rate, and dialysate flow rate (Supplementary Table S3). 9 patients withdrew before the 15th session—7 in the Theranova^®^ 500 group (29.2%) vs. 2 in the Elisio^®^ HX21 group (8.3%), *p* = 0.14. In the Theranova^®^ 500 group, the primary reason for dialyzer disqualification was failure to clear intra-dialyzer blood clots during reprocessing, accounting for 5 of the 7 terminations (2 after the 5th session, 2 after the 8th, and 1 after the 10th session). The remaining 2 patients were withdrawn due to a reduction in TCV > 20%. In the Elisio^®^ HX21 group, 2 terminations were attributable to inability to remove blood clots from the dialyzer (after the 8th and 14th sessions, respectively). The Theranova^®^ 500 group demonstrated a trend toward a lower dialyzer survival rate compared to the Elisio^®^ HX21 group, with an incidence rate of premature termination of 2.24 vs. 0.57 events per 100 person-sessions. Kaplan–Meier analysis revealed a borderline significant difference in survival (log-rank test *p* = 0.06; HR 3.98; 95% CI 0.83–19.20; *p* = 0.085), as shown in Figure 3.

### 3.1. Solute Removal Performance Between the Two Dialyzers

**Figure 4:**
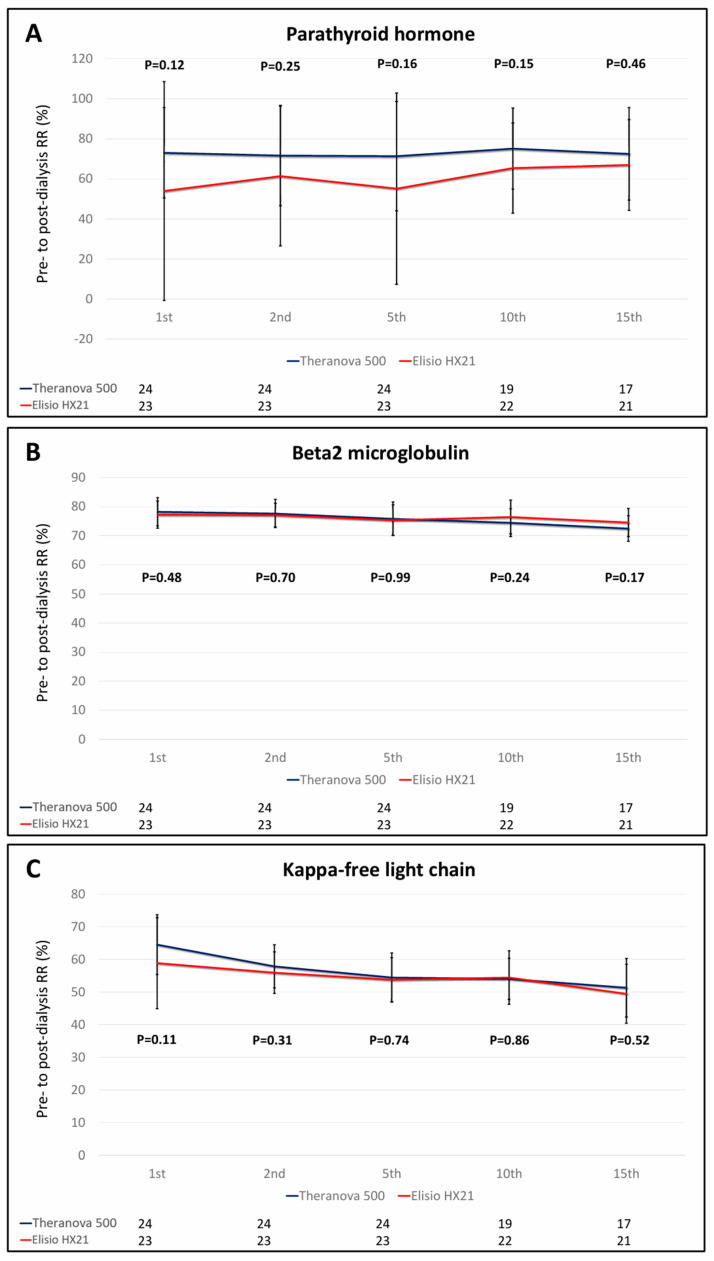

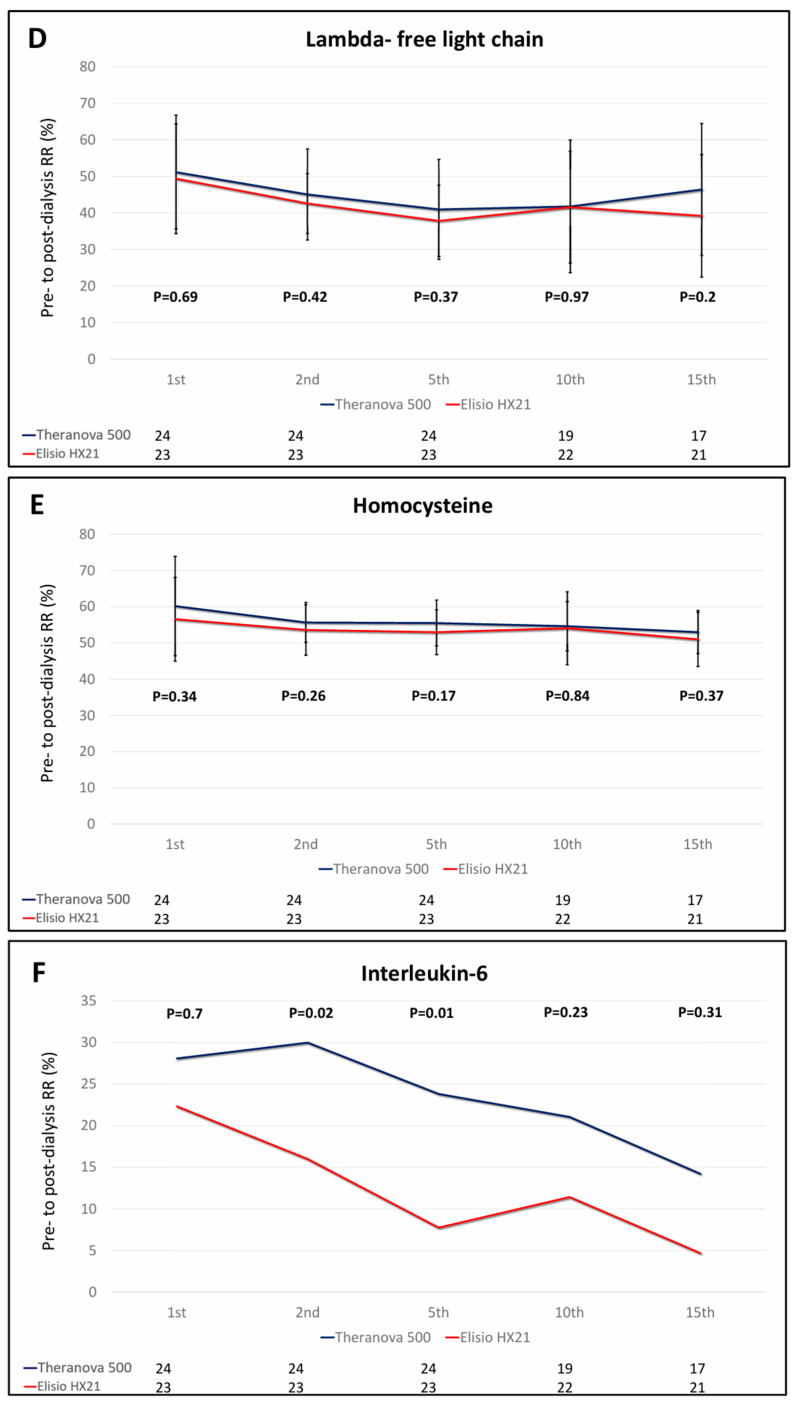

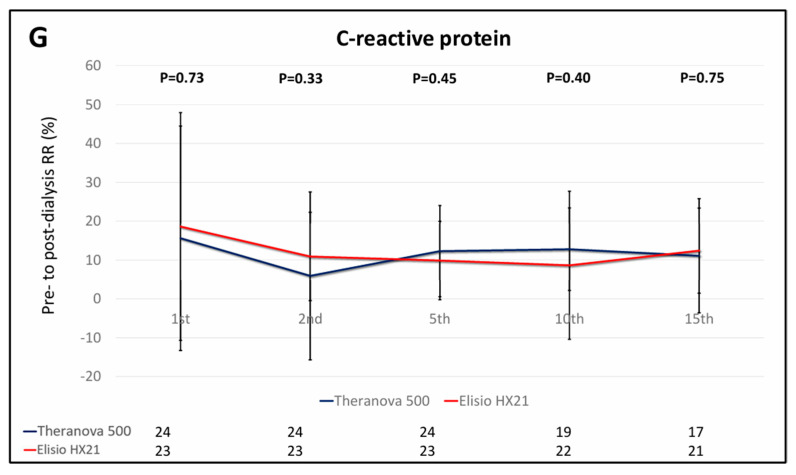
Comparisons of pre- to post-dialysis reduction ratios (RRs) of middle-molecule uremic toxins and inflammatory markers between 2 types of reused MCO dialyzer (Theranova^®^ 500 and Elisio^®^ 21HX). Note: (**A**) Parathyroid hormone (**B**) Beta-2 microglobulin (**C**) Kappa-free light chain (**D**) Lambda- free light chain (**E**) Homocysteine (**F**) Interleukin-6 (**G**) C-reactive protein.

In terms of solute removal performance, the RRs for a range of uremic toxins including urea, phosphate, PTH, β2-MG, κ-FLC, λ-FLC, CRP, and homocysteine revealed comparability between the two groups. This equivalence in removal efficacy was consistent across both the initial and subsequent uses, spanning overall the 2nd, 5th, 10th, and 15th dialysis sessions (Figure 4).

In contrast to the overall similarity in solute removal, IL-6 clearance demonstrated a significant difference between groups at early dialysis sessions. Patients in the Theranova^®^ 500 group exhibited significantly higher IL-6 RRs during the 2nd and 5th sessions compared to those in the Elisio^®^ HX21 group (median IL-6 RR: 29.98% vs. 15.99%, *p* = 0.018; 23.82% vs. 7.74%, *p* = 0.013, respectively). However, no significant differences were observed during the 10th and 15th sessions (median IL-6 RR: 21.05% vs. 11.42%, *p* = 0.23; 14.21% vs. 4.68%, *p* = 0.31). This trend suggests a potential decline in IL-6 clearance efficiency in the Theranova^®^ 500 group with repeated use. Detailed RRs data for uremic toxins and inflammatory markers are presented in Table 2.

The GEE analysis confirmed higher mean IL-6 clearance with Theranova^®^ 500 across all sessions (mean RR difference: 14.67%; 95% CI: 2.14–27.20; *p* = 0.022). No significant between-group differences were found for other solutes (Supplementary Table S4).

### 3.2. Effect of Dialyzer Reuse on Uremic Toxin Removals

The secondary objective was to evaluate the efficacy of uremic toxin removal during initial versus repeated use of the Theranova^®^ 500 and Elisio^®^ HX21 dialyzers. The analysis revealed a progressive decline in clearance efficiency upon reuse, particularly for medium to large middle-molecule uremic toxins. In details, small-molecule solutes such as urea and phosphorus, as well as small middle-molecule toxins PTH and β2-MG, there were no significant reductions in RRs across repeated sessions in either group. An exception was noted only at the 15th dialysis session, where β2-MG clearance showed a significant decrease in both groups (Theranova^®^ 500: from 78.2% to 72.45%, *p* = 0.002; Elisio^®^ HX21: from 77.23% to 74.55%, *p* = 0.006).

In contrast, reductions in clearance were more pronounced for larger middle-molecule solutes. In the Theranova^®^ 500 group, κ-FLC clearance significantly declined from the 2nd session onward (mean RR from 64.55% to 57.89%, *p* < 0.001), and λ-FLC clearance was significantly reduced during the 5th session (mean RR from 51.17% to 40.97%, *p* = 0.02). IL-6 clearance did not show a statistically significant change over time in this group.

In the Elisio^®^ HX21 group, significant declines were noted in κ-FLC RRs during the 5th session (58.88% to 53.79%, *p* = 0.049), λ-FLC RRs from the 2nd session onward (49.39% to 42.59%, *p* = 0.01), and IL-6 RRs during the 15th session (median RR from 22.31% to 4.68%, *p* = 0.01). Additionally, homocysteine clearance significantly declined during the 15th session (mean RR from 56.56% to 50.97%, *p* = 0.003). Detailed data are presented in Table 2.

### 3.3. Safety of the Interventions

**Figure 5:**
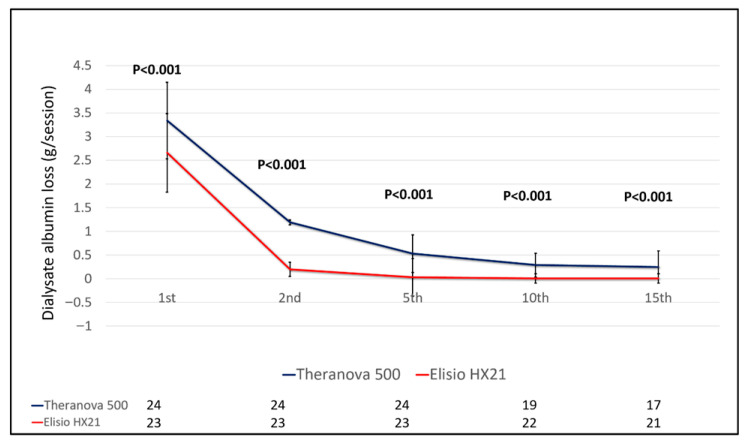
Comparison of total albumin loss in dialysate during hemodialysis using reprocessed dialyzers compared between the Theranova^®^ 500 and Elisio^®^ 21HX groups. Note: There was a significant reduction in dialysate albumin loss after reusing MCO dialyzers (2nd, 5th, 10th, and 15th sessions) when compared with the newly used (1st session) in both Theranova^®^ 500 and Elisio^®^ 21HX groups (*p* < 0.001).

The study demonstrated that the Theranova^®^ 500 group experienced a significantly greater total albumin loss via dialysate compared to the Elisio^®^ HX21 group, both during initial use and across repeated sessions. Albumin loss per session in the Theranova^®^ 500 versus Elisio^®^ HX21 groups at the 1st, 2nd, 5th, 10th, and 15th sessions was as follows: 3.34 ± 0.81 vs. 2.66 ± 0.83 g (*p* = 0.005), 1.19 ± 0.50 vs. 0.20 ± 0.15 g (*p* < 0.001), 0.53 ± 0.40 vs. 0.03 ± 0.04 g (*p* < 0.001), 0.29 ± 0.25 vs. 0.01 ± 0.01 g (*p* < 0.0001), and 0.25 ± 0.34 vs. 0.01 ± 0.01 g (*p* = 0.002), respectively (Figure 5). Using GEE analysis, the Theranova^®^ 500 group showed a significantly higher dialysate albumin loss across all dialysate sessions compared to the Elisio^®^ HX21 group (mean difference: 0.56 g; 95% CI: 0.41–0.71; *p* < 0.001), as shown in Supplementary Table S5. Notably, total albumin loss in dialysate significantly reduced at the second use onwards in both groups.

Despite these differences in dialysate albumin loss, serum pre-dialysis albumin levels at study completion remained stable and were not significantly different from baseline in either group (Theranova^®^ 500: 4.08 ± 0.25 to 4.06 ± 0.26 g/dL; Elisio^®^ HX21: 4.16 ± 0.34 to 4.04 ± 0.29 g/dL).

No adverse events were reported during the study period. Specifically, there were no incidents of intradialytic dialyzer clotting, membrane leakage, severe dialyzer reactions, blood stream infection or hospitalization in either group.

## 4. Discussion

Dialyzer reuse has been an integral component of HD practice since its inception, and its prevalence has notably increased over recent decades in several countries. The proportion of HD centers practicing dialyzer reuse rose markedly from 18% in 1976 to 82% in 1997, although it declined modestly to 63% by 2002. Importantly, the vast majority of centers (94%) conduct dialyzer reprocessing in-house [32]. Previous studies have demonstrated that dialyzer reuse can be safe if conducted in strict accordance with standardized reprocessing protocols. Nonetheless, maintaining rigorous quality control in clinical practice can be challenging. For instance, outbreaks of bloodstream infections have been reported in hemodialysis centers, most likely due to contamination or inadequate disinfection during the reprocessing of reused dialyzers, resulting in increased risks of hospitalization and mortality [33,34]. Conversely, previous reports have demonstrated that the reuse of hemodialyzers—averaging 17 reuses per dialyzer—is safe and effective, with no significant differences observed in mortality, pyrogenic reactions, or infectious complications. In addition, the adoption of dialyzer reuse in the United States has been linked to reductions in patient mortality rates [35]. Similar finding from a systematic review of 14 studies encompassing 956,807 hemodialysis patients reusing dialyzers with chemical disinfectants—including hypochlorite, formaldehyde, glutaraldehyde, and peracetic acid—found that there was no statistically significant difference in mortality between single-use and reused dialyzers [36]. Beyond potential clinical benefits, dialyzer reuse also confers with favorable cost-effectiveness and environmental sustainability.

The impact of reprocessing on dialyzer clearance varies depending on several factors: the type of dialyzer, the reprocessing method employed (including the use of bleach or thermal/chemical disinfectants), the number of reuse cycles, and the class of uremic toxins under consideration (small vs. middle molecules). The efficiency of dialyzers can be influenced by reprocessing practices. Reprocessing without bleach typically leads to a progressive decline in membrane permeability, particularly for larger molecules. In contrast, bleach-based methods may progressively increase permeability to larger molecules, including albumin [22,37,38,39]. Historically, quality control of dialyzer reprocessing has focused primarily on small molecule clearance. However, with the advent of modern dialyzers—such as high-flux and medium cut-off (MCO) dialyzers—greater emphasis must be placed on assessing middle molecule clearance, since these devices are specifically designed to enhance the removal of middle-molecule uremic toxins [16,17,40,41,42]. Previous studies have consistently demonstrated that both low-flux and high-flux hemodialysis (HD) are highly effective in removing small-sized uremic toxins (molecular weight < 500 Da), such as urea and creatinine, irrespective of the reprocessing or disinfection method used. For middle-molecule uremic toxin, HDx using MCO dialyzer provides higher removal performance than high-flux HD [4,43]. However, there are very limited studies on reused MCO at the present time.

To our knowledge, this is the first study to directly compare the efficacy and safety of two reprocessed MCO dialyzers, Theranova^®^ 500 and Elisio^®^ HX21. Using of both types of MCO dialyzers, the clearance of small and medium middle-molecule toxins including urea, phosphate, PTH, β2-microglobulin, κ-FLC, and λ-FLC from MCO dialyzer was consistently maintained, with pre- to post-dialysis RRs exceeding 60% at both first use and after the 15th reuse. In contrast, clearance of large middle-molecule toxins (IL-6 and CRP), which are inherently less effectively removed by MCO membranes, was modest (RRs 15–30%) and declined by more than 20% after the 5th reuse. Protein-bound toxins (Homocysteine), which are intrinsically poorly cleared by MCO membranes, were not affected by reprocessing. It facilitates the routine use of advanced and higher-cost MCO membranes by offsetting expenses. This strategy enables the maintenance of high-quality dialysis care in the face of escalating healthcare expenditures, resource limitations, and fixed reimbursement frameworks.

β2-MG, a small-sized middle-molecule uremic toxin, has been associated with increased mortality in dialysis patients [44]. In the present study, both MCO dialyzers demonstrated comparable efficacy in β2-MG removal, even after multiple reuses. Although a statistically significant reduction in β2-MG RRs was observed during the 15th dialysis session in both groups, the decline from initial use was modest and clinically acceptable—7.23% for Theranova^®^ 500 (from 78.2% to 72.45%) and 3.47% for Elisio^®^ HX21 (from 77.23% to 74.55%). According to standard dialysis guidelines, reprocessed dialyzers are considered acceptable as long as their solute clearance does not decline by more than 10% compared to initial use [25]. Comparison with a previous study reporting that high-flux polysulfone dialyzers reprocessed with peracetic acid showed a decrease in β2-MG clearance from 30% at first use to 12% after ten reuses (a 60% decline), despite preservation of TCV (>80% of initial volume) and stable spKt/V [38].

IL-6 is a medium-sized middle-molecule uremic toxin involved in both acute and chronic inflammatory responses. It is particularly associated with aging-related comorbidities and is recognized as a significant cardiovascular risk marker [45]. In this study, the Theranova^®^ 500 dialyzer demonstrated significantly higher IL-6 reduction ratios (RRs) than the Elisio^®^ 21HX at the 2nd and 5th dialysis sessions, although this difference was not statistically significant during the 10th and 15th sessions. The diminished IL-6 RRs over time may reflect attenuation in membrane performance with repeated use, along with a higher rate of premature termination in the Theranova^®^ 500 group, which might have impacted IL-6 RRs in the later dialysis sessions. A previous in vitro study evaluating the reprocessing of high-flux dialyzers (Polyflux^®^-17R) using a peroxyacetic acid/acetic acid/hydrogen peroxide solution (Renalin^®^) after 0, 1, 5, 10, and 15 reuse sessions—following exposure to endotoxin-contaminated dialysate—found that IL-6 production significantly decreased at reuses 0, 1, 10, and 15 (*p* = 0.03), suggesting membrane adsorption due to reuse-dependent surface binding. However, an in vivo study involving six HD patients demonstrated no significant changes in IL-6 levels over the course of 15 reuse sessions [46]. It was also noted that IL-6 secretion follows a circadian rhythm with daily peaks and troughs and a time variable between days, which could be a confounding factor. Additionally, strong evidence indicates that serum concentrations of IL-6 significantly increase in elderly patients; mean values range from 1.4 pg/mL in males and 1.1 pg/mL in females aged 65–74 years to 3.5 pg/mL in males and 2.1 pg/mL in females aged 85 years and older [47]. Nevertheless, our study observed a reduction in pre-dialysis serum IL-6 levels by the end of the study, decreasing from 1st session levels of 10.58 ± 16.71 to 5.62 ± 2.69 pg/mL (*p* = 0.083) in the Theranova^®^ 500 group and from 7.85 ± 6.20 to 4.88 ± 3.59 pg/mL (*p* = 0.02) in the Elisio^®^ 21HX group, which might reflect the efficacy of MCO dialyzers in IL-6 clearance.

This study demonstrated a significantly greater total dialysate albumin loss with the Theranova^®^ 500 compared to the Elisio^®^ 21HX. However, the safety profile of MCO dialyzers is maintained through controlled pore size design, which restricts albumin loss to below 5 g per session. This precautionary measure is based on the hypothesis that hepatic synthesis can adequately compensate for albumin losses within this range. A prior study involving four types of MCO dialyzers (Phylther^®^ 17-SD (Medtronic Inc., Mirandola, Italy), Vie-18X^®^ (Asahi Kasei Medical Co., Tokyo, Japan), Elisio^®^ 19HX, and Theranova^®^ 400) reported dialysate albumin losses of 1.5–2.0 g/session without significant reductions in serum albumin levels [48]. In the present study, total dialysate albumin loss significantly decreased from the second use onward in both groups. This suggests that the filter membrane’s pore size was reduced following reuse with peracetic acid, representing a potential benefit of dialyzer reuse. Serum albumin levels monitored at the end of the study also showed no significant change from baseline in either group. Previous studies have indicated that dialyzer reuse can alter the membrane structure of polysulfone dialyzers, potentially increasing β2-MG clearance and albumin loss, as well as affecting membrane biocompatibility. However, these studies utilized bleach-based reprocessing, which is thought to contribute to structural membrane alterations [39,49]. Another study investigating extended reuse of polysulfone dialyzers—comparing the 25th use to the 15th—utilizing citric acid and prolonged heat exposure (CAH) found that reprocessing with CAH up to 25 times significantly increased albumin loss and β2-MG clearance, while urea and creatinine clearance remained unaffected [42]. These findings suggest that the impact of dialyzer reuse on albumin loss and uremic toxin clearance may vary based on the reprocessing technique used. Supporting this, a crossover study of polysulfone dialyzers reprocessed with either peracetic acid alone or in combination with bleach found that albumin loss into the dialysate remained clinically insignificant with both reprocessing methods across all sessions. However, β2-MG clearance tended to increase after the seventh reuse in dialyzers reprocessed with peracetic acid and bleach [50].

Reprocessing may also reduce the incidence of dialyzer-related reactions such as “first-use syndrome,” which can occur with new dialyzers due to leaching of substances like ethylene oxide, glycerol, and phthalates [21,22]. However, the reuse process itself involves exposure to chemical agents that pose potential risks to both patients and healthcare staff. Reported complications include allergic reactions, residual chemical infusion (“rebound release”), and pyrogenic reactions. Historically, formaldehyde disinfectant has been associated with hemolysis, as it is metabolized to formic acid, which depletes red blood cell ATP stores [21,22]. Adequate rinsing of disinfected dialyzers to ensure undetectable levels of residual chemicals is therefore a critical step to protect both patients and dialysis personnel [17,40]. In our study, no dialyzer reactions, bloodstream infections, hospitalizations, or deaths occurred during the study period.

From an economic standpoint, dialyzer reuse is particularly relevant in resource-limited settings, where it can reduce dialyzer costs by up to 30%, thereby improving access to hemodialysis [17]. Nevertheless, reprocessing is labor-intensive, increases staff workload, and carries potential occupational risks, which may offset some of the financial benefits. One economic analysis reported direct cost savings of approximately 15% (including medications, dialyzers, consumables, disinfection fluids, and hospitalizations) with reuse [51]. However, the cost difference between single-use and reused dialyzers may vary according to local factors such as dialyzer prices, consumables used in reprocessing, and staff wages [16,17]. Importantly, reprocessing may improve cost-effectiveness by making high-cost dialyzers—such as those with MCO membranes—more affordable, thereby expanding access to HDx and potentially improving patient outcomes. Future studies should focus on cost-utility analyses to more comprehensively evaluate the economic and clinical implications of dialyzer reuse.

Green nephrology emphasizes reducing clinical waste in the care of patients with kidney disease. Dialysis generates substantial waste, primarily from dialyzers, blood tubing sets, syringes, and packaging, amounting to approximately 2 kg per session [52]. A patient undergoing thrice-weekly dialysis without dialyzer reuse produces an estimated 390 kg of waste annually, including 101 kg of polyvinyl chloride (PVC), the predominant material [53]. Such waste poses both biological risks—through infectious or toxic contamination—and environmental hazards, particularly from non-recycled plastics. Implementing dialyzer reuse and promoting plastic recycling are key strategies to minimize dialysis-related waste and advance the principles of green nephrology [52,54,55].

A key strength of this study is the first to demonstrate that HDx using reused MCO membranes up to 15 sessions maintains effective β2-MG removal without compromising patient safety, offering a potential cost-effective strategy for sustainable dialysis care. The limitations of this study include the relatively small sample size, the single-center design, and the number of patients who were prematurely withdrawn due to failed reprocessing, particularly in the Theranova^®^ 500 group, where only 17 of the initial 24 patients completed all 15 dialysis sessions. In addition, the limited duration of this study restricts a meaningful evaluation of long-term clinical outcomes and the potential for adverse effects. Therefore, larger, well-designed multicenter studies with longer follow-up are warranted to rigorously establish the safety and efficacy of reprocessed MCO dialyzers across diverse patient populations.

## 5. Perspectives for Clinical Practice

Our study demonstrates that expanded hemodialysis (HDx) using reused MCO dialyzers, including Theranova^®^ 500 and Elisio^®^ 21HX, maintains efficacy in removing small and medium middle-molecule uremic toxins while ensuring patient safety, even after repeated use. These findings support the feasibility of dialyzer reuse in resource-limited settings, where balancing cost, accessibility, and treatment quality is critical [17,40]. Importantly, we observed that clearance of small and medium middle-molecule uremic toxins (urea, phosphate, PTH, β2-microglobulin, κ-FLC, and λ-FLC) remained within the acceptable range after 15th reuses, without any adverse events associated with dialyzer reprocessing. Based on these findings, we propose that MCO dialyzers can be safely reused for up to 15 sessions while preserving the clinical benefits of expanded hemodialysis, particularly for the removal of middle-molecule toxins associated with inflammation and adverse outcomes.

Dialyzer reuse can enhance dialysis accessibility and sustainability, provided that international guidelines and local quality assurance programs are strictly followed, including careful monitoring of solute clearance, membrane integrity, and microbiological safety [15,24,25,26]. Emerging dialyzer technologies, such as medium and high cut-off membranes, should be rigorously evaluated under reuse conditions to confirm their efficacy and safety [4,41,48]. Furthermore, dialyzer reuse offers potential environmental benefits by reducing biomedical waste, aligning with green dialysis principles that mitigate environmental impact and promote sustainability in healthcare [52,53,54,55]. Implementing evidence-based reuse strategies for MCO dialyzers can therefore optimize patient outcomes while balancing economic and environmental considerations [56,57].

## 6. Conclusions

In conclusion, Theranova^®^ 500 and Elisio^®^ 21HX demonstrated comparable efficacy in removing middle-molecule uremic toxins of varying sizes, including β2-MG, κ-FLC, and λ-FLC, during both initial and repeated uses. While clearance efficiency for certain medium- and large-sized middle molecules declined following dialyzer reuse in both groups, these reductions remained within clinically acceptable limits. Albumin loss into the dialysate was higher with Theranova^®^ 500 but remained within safe thresholds and notably decreased after dialyzer reprocessing in both groups. No serious adverse events were observed during the study period. These findings indicate that HDx using reused MCO dialyzers is both feasible and safe, supporting their clinical utility in resource-limited settings. When implemented with adherence to quality assurance programs, dialyzer reuse can enhance dialysis accessibility and sustainability, optimize patient outcomes, and contribute to environmentally responsible dialysis practices.

## Figures and Tables

**Table 1:** Baseline patient characteristics.

Characteristics	Theranova500 (*n* = 24)	Elisio 21Hx (*n* = 24)	*p*-Value
Age: years (mean ± SD)	63.98 ± 14.89	63.21± 12.66	0.84
Gender: Male *n* (%)	15 (62.5)	15 (62.5)	1
Cause of End-stage kidney disease: (*n*) %			
Diabetic nephropathy	14 (58.33)	12 (50.00)	0.5
Hypertensive nephropathy	2 (8.33)	6 (25)	
Glomerular disease	4 (16.67)	2 (12.5)	
Unknown	1 (4.17)	2 (8.33)	
Other	3 (12.5)	2 (8.33)	
Vascular access: AV fistula, *n* (%)	22 (91.67)	19 (79.17)	0.42
Dialysis vintage: years (mean ± SD)	4.75 ± 3.19	4.01 ± 2.20	0.36
spKt/V: mean ± SD	1.89 ± 0.30	1.87 ± 0.26	0.88
Laboratory: mean ± SD			
- Hemoglobin: (g/dL)	10.73 ± 1.71	10.54 ± 1.13	0.65
- BUN: (mg/dL)	70.59 ± 18.97	70.09 ± 19.26	0.78
- Creatinine: (mg/dL)	11.29 ± 3.81	11.19 ± 2.58	0.83
- Calcium: (mg/dL)	8.50 ± 0.78	8.67 ± 0.71	0.47
- Phosphate: (mg/dL)	4.00 ± 1.78	4.74 ± 1.70	0.15
- Serum albumin: (g/dL)	4.03 ± 0.34	4.14 ± 0.31	0.24
- PTH: (pg/mL)	349.00 ± 265.57	45,451.43 ± 321.18	0.23
- β2-MG: (mg/L)	31.59 ± 8.63	34.78 ± 9.50	0.23

Abbreviation: AV fistula, arteriovenous fistula; spKt/V, single-pool urea Kt/V; BUN, blood urea nitrogen; PTH, parathyroid hormone; β2-MG, beta-2 microglobulin; SD, standard deviation.

**Table 2:** Pre- to post-dialysis reduction ratio (RR) of various-sized uremic toxin and inflammatory markers compared between 2 types of reprocessing MCO dialyzers (Theranova^®^ 500 and Elisio^®^ 21HX) at 1st, 2nd, 5th, 10th, and 15th dialysis sessions.

Uremic Toxins	Dialyzer	Reduction Ratios (%)				
		1st Session	2nd Session	5th Session	10th Session	15th Session
BUN (mean ± SD)	Theranova 500	81.08 ± 4.8	80.5 ± 5.5	80.7 ± 3.1	81.16 ± 5.8	78.53 ± 4.7
	Elisio 21HX	80.67 ± 5.1	81.09 ± 5.6	80.8 ± 4.6	79.23 ± 13.7	80.7 ± 6.3
	*p*-value	0.77	0.72	0.99	0.57	0.24
Phosphate (mean ± SD)	Theranova 500	59.22 ± 10.1	57.46 ± 11.2	58.95 ± 13.3	60.99 ± 13.9	58.28 ± 9.8
	Elisio 21HX	58.25 ± 16.8	56.84 ± 11.3	58.15 ±11.4	60.22 ±12.3	54.73 ±18.9
	*p*-value	0.81	0.85	0.82	0.85	0.49
PTH (mean ± SD)	Theranova 500	73.02 ± 22.6	71.65 ± 25.1	71.34 ± 27.3	75.11 ± 20.2	72.49 ± 23.1
	Elisio 21HX	53.96 ± 54.6	61.39 ± 34.9	55.12 ± 47.8	65.4 ± 22.5	66.96 ± 22.7
	*p*-value	0.12	0.25	0.16	0.15	0.46
β2-MG (mean ± SD)	Theranova 500	78.22 ± 4.9	77.61 ± 4.9	75.82 ± 5.7 *	74.45 ± 4.7	72.45 ± 4.4 *
	Elisio 21HX	77.23 ± 4.7	77.1 ± 4.1	75.33 ± 5.3	76.43 ± 5.8	74.55 ± 4.8 *
	*p*-value	0.48	0.69	0.99	0.24	0.17
κ-FLC (mean ± SD)	Theranova 500	64.55 ± 9.2	57.89 ± 6.6 *	54.48 ± 7.5 *	54.05 ± 6.3 *	51.33 ± 8.9 *
	Elisio 21HX	58.88 ± 13.9	55.96 ± 6.4	53.79 ± 6.7 *	54.46 ± 8.2	49.48 ± 9.0
	*p*-value	0.11	0.31	0.74	0.86	0.53
λ-FLC (mean ± SD)	Theranova 500	51.17 ± 15.6	45.07 ± 12.5	40.97 ± 13.7 *	41.79 ± 18.1	46.43 ± 18.0 *
	Elisio 21HX	49.39 ± 15.0	42.59 ± 8.2 *	37.85 ± 9.8 *	41.59 ± 15.3 *	39.21 ± 16.8 *
	*p*-value	0.69	0.42	0.37	0.97	0.20
IL-6 Median (IQR)	Theranova 500	28.08	29.98	23.82	21.05	14.21
		(7.3, 37.4)	(18.7, 41.6)	(11.0, 34.2)	(5.7, 35.4)	(−3.5, 35.4)
	Elisio 21HX	22.32	15.99	7.74	11.42	4.68 *
		(1.12, 37.7)	(−16.2, 28.0)	(−18.9, 27.8)	(−17.7, 34.6)	(−21.4, 31.2)
	*p*-value	0.7	0.018	0.013	0.231	0.31
CRP (mean ± SD)	Theranova 500	15.59 ± 28.9	5.91 ± 21.6	12.27 ± 11.7	12.77 ± 10.6	11.08 ± 14.7
	Elisio 21HX	18.59 ± 29.3	10.9 ± 11.4	9.85 ± 10.1	8.63 ± 19.1	12.41 ± 10.9
	*p*-value	0.73	0.33	0.45	0.40	0.75
Homocysteine (mean ± SD)	Theranova 500	60.17 ± 13.7	55.64 ± 5.5	55.49 ± 6.3	54.62 ± 6.8	52.97 ± 5.9
	Elisio 21HX	56.56 ± 11.5	53.57 ± 7.0	52.95 ± 6.2	54.06 ± 10.1	50.97 ± 7.4 *
	*p*-value	0.33	0.26	0.16	0.83	0.37

Note: * Significantly reduction in pre- and post-dialysis RR compared with newly used dialyzer in 1st session (*p* < 0.05). Abbreviation: CRP, C-reactive protein; β2-MG, beta-2 microglobulin; IL-6, interleukin-6; κ-FLC, kappa free light chain; λ-FLC, lambda free light chain; IQR, interquartile range; SD, standard deviation.

## Data Availability

The original contributions presented in this study are included in the article/Supplementary Material. Further inquiries can be directed to the corresponding author.

## Supplementary Materials

The following are available online at https://www.mdpi.com/article/10.3390/life15091468/s1, Supplementary Methodology Dialyzer reprocessing instructions; Supplementary Figure S1. Flow-diagram of patient enrollment; Supplementary Table S1. Dialyzer characteristics; Supplementary Table S2. Bacterial culture and endotoxin levels of dialysis water and dialysate during the study period; Supplementary Table S3. Comparison of dialysis intervention in the study sessions; Supplementary Table S4. The overall mean difference in reduction ratios for solute removals and total dialysate albumin loss between the two groups assessed by the generalized estimating equation; Supplementary Table S5. Pre- and Post-dialysis level of various-sized uremic toxin and inflammatory markers compared between 2 types of reprocessing MCO dialyzers (Theranova® 500 and Elisio® 21HX) at 1st, 2nd, 5th, 10th, and 15th dialysis sessions; Supplementary Table S6. Legend of Acronyms in manuscript; CONSORT 2025 Check list for Randomized control clinical study. Ref. [58] was cited in Supplementary Materials.
