# Supplementary material for: Comparative Efficacy of Reused Medium Cut-Off Dialyzers on Uremic Toxin and Cytokine Clearance: A Randomized Controlled Trial
Source: Life (Basel). 2025 Sep 18;15(9):1468. doi: 10.3390/life15091468 (PMC12472171; doi:10.3390/life15091468)
Supplement: Supplementary file 1 [file life-15-01468-s001.zip › Life-3826615-Supplementary materials.pdf]

Comparative Efficacy of Reused Medium Cut-Off Dialyzers on Uremic Toxin and Cytokine Clearance: A Randomized Con-trolled Trial.

Eakalak Lukkanalikitkul, Nichnan Jirayuphat, Sirirat Anutrakulchai

Supplementary Materials

Supplementary Methodology Dialyzer reprocessing instructions

Supplementary Figure S1. Flow-diagram of patient enrollment

Supplementary Table S1. Dialyzer characteristics

Supplementary Table S2. Bacterial culture and endotoxin levels of dialysis water and dialysate during the study period

Supplementary Table S3. Comparison of dialysis intervention in the study sessions

Supplementary Table S4. The overall mean difference of reduction ratios for solute removals and total dialysate albumin loss between the two groups assessed by the generalized estimating equation.

Supplementary Table S5. Pre- and Post-dialysis level of various-sized uremic toxin and inflammatory markers compared between 2 types of reprocessing MCO dialyzers (Theranova® 500 and Elisio® 21HX) at 1st, 2nd, 5th, 10th, and 15th dialysis sessions.

Supplementary Table S6. Legend of Acronyms in manuscript

CONSORT 2025 Check list for Randomized control clinical study

Dialyzer reprocessing instructions

This protocol outlines the standard procedure for the manual cleaning, disinfection, and reuse of dialyzers and bloodlines at Srinagarind Hospital, Faculty of Medicine, Khon Kaen University.

1. Post-dialysis handling

- After completion of a dialysis session, ensure all connectors of the used dialyzer are securely closed, the bloodlines are clamped, and the ends are capped.
- Transport the dialyzer and associated bloodlines into a leak-proof container to the designated washing station.

2. Personal protective equipment (PPE)

- Prior to reprocessing, all personnel involved must wear appropriate PPE, including:
    - Goggles or face shield
    - Surgical or N95 mask
    - Hair cap
    - Long-sleeved plastic gown
    - Gloves

3. Preliminary cleaning of bloodlines

- Rinse one end of the bloodline with reverse osmosis (RO) water until visibly clear of blood residue, keeping the other end clamped to prevent splashing or contamination.

4. Disconnection and cleaning

- Disconnect the bloodline from the dialyzer.
- Clean all components (bloodline and dialyzer exterior) with RO water.
- Use a rubber mallet to gently tap and remove any clotted blood or fibrin deposits within the dialyzer.

5. Dialyzer compartment cleaning

- Clean both the blood and dialysate compartments with pressurized RO water for 3–10 minutes at less than 15 pounds per square inch.
- Reverse the water flow direction periodically to simulate ultrafiltration.
- For dialyzers with removable caps, manually clean the internal surfaces.
- Reinspect all parts to ensure complete removal of contaminants.

6. Total Cell Volume (TCV) measurement

- Connect both the blood and dialysate compartments, then fill the dialyzer with RO water until fully saturated.
- Seal the dialysate compartment and use a pressure bulb to push water from the blood compartment into a calibrated measuring cylinder.
- Measure the TCV and compare it to the original priming volume.
    - If TCV ≥ 80%, proceed to disinfection.
    - If TCV < 80%, discard the dialyzer.

7. Dialyzer leak test

- Drain all water from both compartments.
- Connect a pressure gauge to the blood compartment and increase the internal pressure to 250 mmHg.
- Clamp and observe for 30 seconds.
- If pressure drops ≥ 25 mmHg (low-flux dialyzer) or ≥ 40 mmHg (high-flux dialyzer), retest once.
- If the dialyzer fails the second test, it must be discarded.

8. Reconnection and Filling

- Reconnect the dialyzer to the bloodline.
- Fill the blood compartment with RO water until there are no air bubbles present.
- Clamp all branches of the bloodline securely.

9. Disinfectant preparation

- Prepare 1500 mL of disinfectant solution using RO water to 4% peracetic acid mixture (PAM)

10. Internal disinfection

- Connect the bloodline and open the clamp to allow disinfectant solution into the blood compartment—at a volume at least three times that of the dialyzer and bloodline combined.
- Fill the dialysate compartment with disinfectant as well.
- Clamp all ports and verify that both compartments are completely filled, with no air bubbles present.

11. External disinfection

- Immerse the exterior of the dialyzer in 0.05% sodium hypochlorite solution for at least 10 seconds.

- Allow the dialyzer to dry before storage.

12. Storage conditions

- Place the reprocessed dialyzer in a container clearly labeled with the patient’s name.
- Store it in a clean, enclosed area protected from heat and light, maintaining a temperature between 25–27°C.

13. Sterilization validation and storage duration

- Prior to reuse, confirm that sterilization is complete in accordance with standardized times (more than 11 hours under room temperature 25 °C).
- Storage duration post-sterilization up to 1 weeks with 4% PAM.

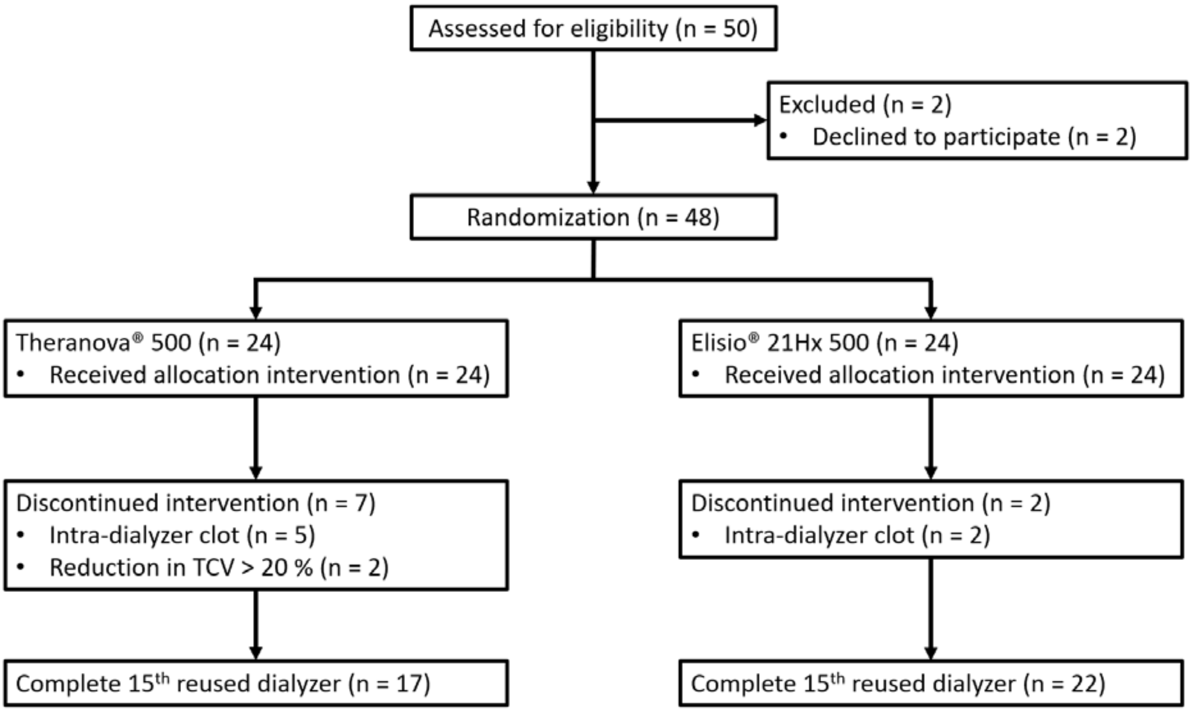

Figure S1. Flow-diagram of patient enrollment

Table S1. Dialyzer characteristics

Characteristics	Theranova 500®	Elisio Hx21®
Membrane	Polyarylethersulfone (PAES)	Polyethersulfone (PES)
Surface area	2.0 m^2	2.1 m^2
Priming volume	105 mL	125 mL
Fiber inner diameter (µm)	180	200
Membrane thickness (µm)	35	40
Effective length (mm)	260	290
Sterilization	Steam	Gamma Ray
KUF (mL/hour/mmHg)	59	82
Clearance (mL/min)		
- Urea	285	292
- Creatinine	274	283
- Phosphate	267	271
Sieving coefficient		
- Vitamin B12	1.0	1.0
- Inulin	1.0	0.97
- β2-MG	1.0	1.0
- Myoglobin	0.9	0.37
- Albumin	0.008	0.0015
Sieving profile		
- MWCO (kDa)	56 ± 3	NA
- MWRO (kDa)	9.4 ± 0.2	NA

Abbreviation: β2-MG, beta-2 microglobulin; MWCO, molecular weight cut-off; MWRO, molecular weight retention onset; N/A, non-available.

Table S2. Bacterial culture and endotoxin levels of dialysis water and dialysate during the study period

	January 2024	February 2024	Mar 2024
Dialysis water			
- Bacterial culture (CFU/ml)	< 2.0	9.0	< 1.0
- Endotoxin level (EU/ml)	< 0.03	0.00290	0.00341
Dialysate fluid			
- Bacterial culture (CFU/ml)*	< 2.0	< 1.0	< 1.0
- Endotoxin level (EU/ml)	< 0.03	0.00447	< 0.004

Note: Bacterial culture was performed by using spread plate method with Trypticase soy agar (TSA) and endotoxin level was performed by using Limulus Amebocyte Lysate (LAL) kinetic turbidimetric method.

Table S3. Comparison of dialysis intervention in the study sessions

	**Theranova500 (mean± SD)**	**Elisio 21Hx (mean± SD)**	**P value**
**Effective dialysis time, (min/session)**	240	240	1.0
**Actual blood flow rate, (mL/min)**	325 ± 39.0	322.1 ± 38.4	0.79
**Actual dialysis flow rate, (mL/min)**	712.5 ± 139.3	727.1 ± 134.3	0.71

Table S4. The overall mean difference of reduction ratios for solute removals and total dialysate albumin loss between the two groups assessed by the generalized estimating equation.

**Parameters**	**Overall mean differences (95% CI) (Theranova - Elisio 21HX levels)**	**P-value**
**Reduction ratio (%)**		
Urea	0.13 (-2.47 to 2.73)	0.92
Creatinine	-0.16 (-2.80 to 2.48)	0.91
Phosphate	1.71 (-4.45 to 7.88)	0.59
Albumin	1.84 (-2.71 to 6.40)	0.43
PTH	12.64 (-3.63 to 28.92)	0.13
β2-MG	-0.08 (2.39 to 2.23)	0.95
κ-FLC	2.66 (-0.77 to 6.10)	0.13
λ-FLC	2.99 (-2.67 to 8.64)	0.30
IL-6	14.67 (2.14 to 27.20)	0.022
CRP	-0.60 (-7.35 to 6.16)	0.86
Homocysteine	2.65 (-1.04 to 6.34)	0.16
**Total dialysate albumin loss (g)**	0.56 (0.41 to 0.71)	< 0.001

Abbreviation: PTH, parathyroid hormone; β2-MG, beta-2 microglobulin; κ-FLC, kappa free light chain; λ-FLC, lambda free light chain; IL-6, interleukin-6; CRP, C-reactive protein

Table S5. Pre- and Post-dialysis level of various-sized uremic toxin and inflammatory markers compared between 2 types of reprocessing MCO dialyzers (Theranova® 500 and Elisio® 21HX) at 1st, 2nd, 5th, 10th, and 15th dialysis sessions.

Uremic toxin	Dialyzer	1st session		2nd session		5th session		10th session		15th session	
		Pre	Post	Pre	Post	Pre	Post	Pre	Post	Pre	Post
BUN (mg/dL)	Theranova 500	70.6±19.0	13.0±3.8	58.0±19.9	10.8±3.5	69.3±18.9	13.0±3.3	62.6±29.8	11.8±6.2	66.8±34.5	14.3±7.9
(mean ± SD)	Elisio 21HX	70.1±19.3	13.1±3.9	59.2±19.2	10.5±3.0	66.5±17.9	12.3±2.7	64.9±22.7	12.8±5.6	62.2±23.3	12.5±5.2
	p value	0.93	0.95	0.83	0.77	0.60	0.42	0.68	0.71	0.40	0.22
Cr (mg/dL)	Theranova 500	11.3±3.8	2.9±1.1	10.3±3.5	2.7±0.9	11.0±4.0	3.0±1.1	10.1±5.1	2.5±1.5	10.3±5.4	2.9±1.6
(mean ± SD)	Elisio 21HX	11.2±2.6	3.0±0.9	10.1±2.2	2.6±0.6	10.6±2.6	2.8±0.6	11.0±3.1	2.8±1.1	10.6±3.8	2.7±1.1
	p value	0.92	0.80	0.81	0.65	0.68	0.55	0.31	0.39	0.73	0.49
Phosphate	Theranova 500	4.1±1.6	1.6±0.5	4.0±2.0	1.6±0.5	4.3±1.8	1.6±0.4	3.8±1.2	1.4±0.6	3.8±1.3	1.6±0.7
(mg/dL)	Elisio 21HX	4.1±1.2	1.7±0.5	4.0±1.2	1.6±0.4	4.3±1.5	1.7±0.4	4.1±1.2	1.6±0.5	3.8±1.2	1.6±0.5
(mean ± SD)	p value	0.98	0.51	0.98	0.48	0.97	0.45	0.30	0.18	0.89	0.94
PTH (pg/mL)	Theranova 500	353±257	107±106	332±267	111±134	356±280	108±108	333±190	82±42	319±195	101±134
(mean ± SD)	Elisio 21HX	362±246	228±304	362±260	199±271	390±344	249±353	463±525	224±339	403±532	218±393
	p value	0.89	0.07	0.70	0.16	0.71	0.07	0.31	0.08	0.54	0.25
β2-MG	Theranova 500	33.0±10.8	8.9±2.6	30.3±8.9	8.3±2.2	32.7±10.7	9.9±3.1	29.5±7.4	9.3±2.5	29.7±7.3	10.2±2.6
(mg/L)	Elisio 21HX	32.5±7.8	9.3±2.3	31.6±6.8	8.8±2.2	33.2±7.9	9.8±3.1	33.6±9.4	9.5±2.3	30.4±6.1	9.5±2.2
(mean ± SD)	p value	0.87	0.62	0.56	0.37	0.87	0.92	0.13	0.78	0.74	0.35
κ-FLC (mg/L)	Theranova 500	297±106.4	145±61	277±95	146±59	285±93	166±63	300±92	170±56	325±100	197±64
(mean ± SD)	Elisio 21HX	281±88	156±73	274±112	151±73	287±116	167±82	337±124	191±82	331±131	210±101
	p value	0.58	0.60	0.92	0.77	0.95	0.99	0.29	0.34	0.88	0.66
λ-FLC (mg/L)	Theranova 500	256±123	172±96	232±110	160±80	228±99	175±87	251±100	177±82	295±80	198±85
(mean ± SD)	Elisio 21HX	261±116	181±94	253±122	184±100	251±115	197±104	299±126	219±116	309±129	233±120
	p value	0.89	0.76	0.53	0.36	0.46	0.43	0.18	0.19	0.68	0.32
IL-6 (pg/ml)	Theranova 500	10.6±16.7	7.9±4.9	8.1±7.3	7.5±7.6	7.5±5.5	7.5±5.1	6.4±4.6	6.6±5.9	5.6±2.7	6.0±3.1
(mean ± SD)	Elisio 21HX	7.8±6.2	7.1±4.4	5.2±3.7	5.4±3.3	7.2±6.6	7.5±5.9	6.9±5.3	7.7±6.1	4.9±3.6	5.6±3.9
	p value	0.46	0.58	0.09	0.21	0.89	0.99	0.73	0.58	0.48	0.72
CRP (mg/L)	Theranova 500	11.7±21.7	6.6±11.1	7.3±13.9	8.4±16.0	4.0±5.5	4.6±6.7	4.0±8.8	4.3±8.7	1.7±1.7	2.0±2.0
(mean ± SD)	Elisio 21HX	5.9±11.0	6.8±11.1	4.0±4.8	4.3±4.9	5.7±9.5	6.3±10.6	3.5±3.2	4.0±3.5	3.1±3.5	3.5±4.1
	p value	0.26	0.93	0.27	0.23	0.45	0.51	0.80	0.90	0.15	0.17
Homocysteine	Theranova 500	29.3±10.2	15.7±5.1	23.9±7.3	13.2±4.3	25.6±8.3	14.6±5.2	23.6±6.8	13.1±3.7	24.2±5.9	14.3±4.0
(U/L)	Elisio 21HX	33.0±5.5	19.3±4.5	27.0±4.4	15.4±3.3	28.0±6.7	16.3±4.5	27.5±4.9	15.6±4.4	27.7±7.3	16.7±4.5
(mean ± SD)	p value	0.14	0.02	0.08	0.046	0.28	0.24	0.04	0.055	0.12	0.09

Abbreviation: Cr; creatinine, CRP, C-reactive protein; β2-MG, beta-2 microglobulin; IL-6, interleukin-6; κ-FLC, kappa free light chain; λ-FLC, lambda free light chain; IQR, interquartile range; SD, standard deviation

Table S6. Legend of Acronyms in manuscript

Acronyms	Description
AVF	Arteriovenous fistula
AVG	Arteriovenous graft
spKt/V	Single-pool urea Kt/V
BUN	Blood urea nitrogen
PTH	Parathyroid hormone
β2-MG	Beta-2 microglobulin
CRP	C-reactive protein
IL-6	Interleukin-6
κ-FLC	Kappa free light chain
λ-FLC	Lambda free light chain
SD	Standard deviation
IQR	Interquartile range
MCO	Medium cut-off dialyzers
ESKD	End-stage kidney disease
HD	Hemodialysis
PBUTs	Protein-bound uremic toxins
HDx	Expanded hemodialysis
TCV	Total cell volume
RRs	Reduction ratios

Section/topic	No	CONSORT 2025 checklist item description	Reported on page no.
**Title and abstract**			
Title and structured abstract	1a	Identification as a randomised trial	1
	1b	Structured summary of the trial design, methods, results, and conclusions	1
**Open science**			
Trial registration	2	Name of trial registry, identifying number (with URL) and date of registration	15
Protocol and statistical analysis plan	3	Where the trial protocol and statistical analysis plan can be accessed	15
Data sharing	4	Where and how the individual de-identified participant data (including data dictionary), statistical code and any other materials can be accessed	15
Funding and conflicts of interest	5a	Sources of funding and other support (eg, supply of drugs), and role of funders in the design, conduct, analysis and reporting of the trial	15
	5b	Financial and other conflicts of interest of the manuscript authors	15
**Introduction**			
Background and rationale	6	Scientific background and rationale	2-3
Objectives	7	Specific objectives related to benefits and harms	3
**Methods**			
Patient and public involvement	8	Details of patient or public involvement in the design, conduct and reporting of the trial	3
Trial design	9	Description of trial design including type of trial (eg, parallel group, crossover), allocation ratio, and framework (eg, superiority, equivalence, non-inferiority, exploratory)	3-4
Changes to trial protocol	10	Important changes to the trial after it commenced including any outcomes or analyses that were not prespecified, with reason	N/A
Trial setting	11	Settings (eg, community, hospital) and locations (eg, countries, sites) where the trial was conducted	3-4
Eligibility criteria	12a	Eligibility criteria for participants	3
	12b	If applicable, eligibility criteria for sites and for individuals delivering the interventions (eg, surgeons, physiotherapists)	3
Intervention and comparator	13	Intervention and comparator with sufficient details to allow replication. If relevant, where additional materials describing the intervention and comparator (eg, intervention manual) can be accessed	4
Outcomes	14	Prespecified primary and secondary outcomes, including the specific measurement variable (eg, systolic blood pressure), analysis metric (eg, change from baseline, final value, time to event), method of aggregation (eg, median, proportion), and time point for each outcome	4-5
Harms	15	How harms were defined and assessed (eg, systematically, non-systematically)	5
Sample size	16a	How sample size was determined, including all assumptions supporting the sample size calculation	3
	16b	Explanation of any interim analyses and stopping guidelines	N/A
Randomisation:			
Sequence generation	17a	Who generated the random allocation sequence and the method used	4
	17b	Type of randomisation and details of any restriction (eg, stratification, blocking and block size)	4
			**Reported on page no.**

Allocation concealment mechanism	18	Mechanism used to implement the random allocation sequence (eg, central computer/telephone; sequentially numbered, opaque, sealed containers), describing any steps to conceal the sequence until interventions were assigned	N/A (open-label)
Implementation	19	Whether the personnel who enrolled and those who assigned participants to the interventions had access to the random allocation sequence	4
Blinding	20a	Who was blinded after assignment to interventions (eg, participants, care providers, outcome assessors, data analysts)	4
	20b	If blinded, how blinding was achieved and description of the similarity of interventions	4-5
Statistical methods	21a	Statistical methods used to compare groups for primary and secondary outcomes, including harms	5-6
	21b	Definition of who is included in each analysis (eg, all randomised participants), and in which group	4-5
	21c	How missing data were handled in the analysis	N/A
	21d	Methods for any additional analyses (eg, subgroup and sensitivity analyses), distinguishing prespecified from post hoc	N/A
**Results**			
Participant flow, including flow diagram	22a	For each group, the numbers of participants who were randomly assigned, received intended intervention, and were analysed for the primary outcome	6
	22b	For each group, losses and exclusions after randomisation, together with reasons	6
Recruitment	23a	Dates defining the periods of recruitment and follow-up for outcomes of benefits and harms	3
	23b	If relevant, why the trial ended or was stopped	N/A
Intervention and comparator delivery	24a	Intervention and comparator as they were actually administered (eg, where appropriate, who delivered the intervention/comparator, how participants adhered, whether they were delivered as intended (fidelity))	4-5
	24b	Concomitant care received during the trial for each group	4-5
Baseline data	25	A table showing baseline demographic and clinical characteristics for each group	6-7
Numbers analysed, outcomes and estimation	26	For each primary and secondary outcome, by group:<br>● the number of participants included in the analysis<br>● the number of participants with available data at the outcome time point<br>● result for each group, and the estimated effect size and its precision (such as 95% confidence interval)<br>● for binary outcomes, presentation of both absolute and relative effect size	6-10
Harms	27	All harms or unintended events in each group	11
Ancillary analyses	28	Any other analyses performed, including subgroup and sensitivity analyses, distinguishing pre-specified from post hoc	N/A
**Discussion**			
Interpretation	29	Interpretation consistent with results, balancing benefits and harms, and considering other relevant evidence	11-14
Limitations	30	Trial limitations, addressing sources of potential bias, imprecision, generalisability, and, if relevant, multiplicity of analyses	14

Citation: Hopewell S, Chan AW, Collins GS, Hróbjartsson A, Moher D, Schulz KF, et al. CONSORT 2025 Statement: updated guideline for reporting randomised trials. BMJ. 2025; 388:e081123. https://dx.doi.org/10.1136/bmj-2024-081123
© 2025 Hopewell et al. This is an Open Access article distributed under the terms of the Creative Commons Attribution License (https://creativecommons.org/licenses/by/4.0/), which permits unrestricted use, distribution, and reproduction in any medium, provided the original work is properly cited.

*We strongly recommend reading this statement in conjunction with the CONSORT 2025 Explanation and Elaboration and/or the CONSORT 2025 Expanded Checklist for important clarifications on all the items. We also recommend reading relevant CONSORT extensions. See www.consort-spirit.org.
